# Quantum Dot Nanobeads as Multicolor Labels for Simultaneous Multiplex Immunochromatographic Detection of Four Nitrofuran Metabolites in Aquatic Products

**DOI:** 10.3390/molecules27238324

**Published:** 2022-11-29

**Authors:** Xiuying Liu, Yuanyuan Cheng, Binbin Guan, Fei Xia, Ling Fan, Xue Gao, Xiaofei Sun, Xuepeng Li, Lijie Zhu

**Affiliations:** 1 College of Food Science and Technology, Bohai University, Jinzhou 121013, China; 2 National & Local Joint Engineering Research Center of Storage, Processing and Safety Control Technology for Fresh Agricultural and Aquatic Products, Jinzhou 121013, China

**Keywords:** nitrofuran metabolites, multiplex immunochromatographic assay, quantum dot nanobeads, fluorescent test strip

## Abstract

A multicolor immunochromatographic assay platform based on quantum dot nanobeads (QBs) for the rapid and simultaneous detection of nitrofuran metabolites in different aquatic products is documented. These metabolites include 3-amino-2-oxazolidinone (AOZ), 1-aminohydantoin (AHD), semicarbazide (SEM), and 3-amino-5-morpholino-methyl-1,3-oxazolidinone (AMOZ). QBs with emission colors of red, yellow, green, and orange were employed and functionalized with the corresponding antibodies to each analyte to develop a multicolor channel. The visual detection limits (cutoff values) of our method for AOZ, AHD, SEM, and AMOZ reached up to 50 ng/mL, which were 2, 20, 20, and 20 times lower than those of traditional colloidal gold test strips, respectively. The test strip is capable of detection within 10 min in real samples while still achieving good stability and specificity. These results demonstrate that the developed multicolor immunochromatographic assay platform is a promising technique for multiplex, highly sensitive, and on-site detection of nitrofuran metabolites.

## 1. Introduction

Nitrofurans are broad-spectrum antibiotics that have been widely used in animal husbandry and aquaculture for the prevention and treatment of bacterial diseases [1]. However, the abuse of nitrofurans may lead to the accumulation of their residues in animal-derived food and in the human body through the food chain. This may result in carcinogenic and mutagenic effects along with antibiotic resistance, thereby threatening the health of animals and humans [2]. To ensure food safety, nitrofurans have been prohibited in many countries, including the European Union, the United States, and China [3]. Nitrofurans are extremely unstable and metabolize rapidly in vivo. However, their metabolites, 3-amino-2-oxazolidinone (AOZ), 1-aminohydantoin (AHD), semicarbazide (SEM), and 3-amino-5-morpholino-methyl-1,3-oxazolidinone (AMOZ), are stable for significant durations because of their binding with proteins, which are generally used to monitor the residues of nitrofurans [4].

To date, various detection platforms have been used to monitor nitrofuran metabolite residues, including high-performance liquid chromatography [5] and liquid chromatography–tandem mass spectrometry [6]. Although these methods exhibit high sensitivity and accuracy, they have disadvantages, including cost, time, and instrumentation requirements. This limits their application for on-site detection. Therefore, it is of great interest to develop a simple, rapid, and sensitive method for the on-site detection of nitrofuran metabolites.

Immunochromatographic assays (ICAs) have unique advantages, such as simplicity, rapidness, low cost, and ease of use. They have been widely employed for on-site detection [7,8]. In an ICA, label signal reporters are important because of detection sensitivity and selectivity [9]. To date, commercially available test strips have been developed based on colloidal gold nanoparticles as reporters for colorimetric detection. However, gold nanoparticle test strips have limited detection sensitivity and lack quantitative detection capability [10]. Recently, fluorescent nanomaterials have attracted significant research interest in the field of analysis and detection because of their high luminescence [11]. Moreover, fluorescent nanomaterials, such as quantum dots (QDs) [12], up-conversion nanoparticles [13], and organic dye microspheres [14], have been introduced as alternatives to gold nanoparticles to improve the ICA’s sensitivity and quantitative detection capability. Among these materials, QDs are considered ideal for fluorescence immunoassays because of their excellent fluorescent properties, including high quantum yields, broad excitation range at narrow emission spectra, strong luminescence, and excellent resistance to light bleaching [15]. A few research groups have reported QD-based ICA methods for the rapid detection of protein biomarkers and other small-molecule compounds and achieved high sensitivity. For example, Wang et al. [16] selected CdSe/ZnS QDs as a fluorescence label to conjugate with antibodies to prepare an immunochromatographic test strip for the rapid detection of the crustaceous major allergen tropomyosin. However, to further improve the visibility of detection results and enhance the application potential of naked-eye detection, numerous QDs were embedded into polymer carriers to construct quantum dot nanobeads (QBs). QBs have strong luminescence, high stability in complex matrices, and good biocompatibility. Consequently, they demonstrate better performance in fluorescent probe labeling [17,18]. However, the fluorescence immunochromatography that has been reported mainly focuses on the detection of single target analytes [19,20]. Thus, multiple immunochromatographic technologies need to be developed to realize multi-residue detection of hazardous substances.

Accordingly, in this study, we developed a new multicolor mICA platform based on QB probes with different emission colors for the rapid and simultaneous detection of nitrofuran metabolites in different aquatic products. The detection sensitivity of the mICA platform can be improved by using QBs as fluorescent labels. Moreover, the feasibility of the proposed mICA platform is demonstrated.

## 2. Results and Discussion

### 2.1. Principle of the QB-Based Multiplex Fluorescent Lateral Flow Immunoassay

**Figure 1:**
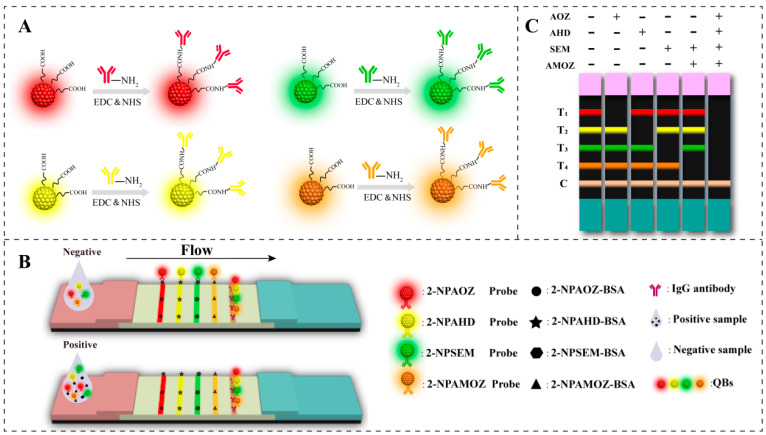
Schematic illustration of the (**A**) preparation of QB-mAb probes, (**B**) analysis of QB-based mICA, and (**C**) interpretation of test results. (“+” and “−” indicate the “positive” and “negative” results.).

The principle of fluorescent mICA used in this study is illustrated in Figure 1. It is based on the competitive integration reaction of antibody–antigen and the fluorescent signal of QBs. As shown in Figure 1A, four types of QBs with distinct emission colors (red, green, yellow, and orange) were selected for labeling the antibodies of the four nitrofuran metabolites. This permitted the development of an mICA for the simultaneous detection of nitrofuran metabolites on a single test strip. Figure 1B shows the analysis process of the QB-based mICA. When the concentrations of the target analyte in the sample extract were below the threshold, the free QB-mAb probes interacted with target analyte–BSA conjugates that were immobilized on the test line; hence, the clear fluorescent signal of the QBs was shown on the test lines. Correspondingly, the excess of QB-mAb probes was subsequently trapped by the specific secondary goat anti-mouse IgG on the C line. As a result, as shown in Figure 1C, the development of fluorescent bands in T1, T2, T3, T4, and C lines indicated a negative result for the four nitrofuran metabolites. When the concentrations of target analytes in the sample exceeded the threshold, the QB-mAb probes first interacted with target analytes. This led to lower or no fluorescent signaling on the test line, indicating a positive result for target analytes in the sample extract. The T1, T2, T3, and T4 lines represent the target analytes of AOZ, AHD, SEM, and AMOZ, respectively. The weakening or disappearance of the corresponding bands indicates that the corresponding target analytes were positive.

### 2.2. Characterization of QBs and QB-mAb Probes

**Figure 2:**
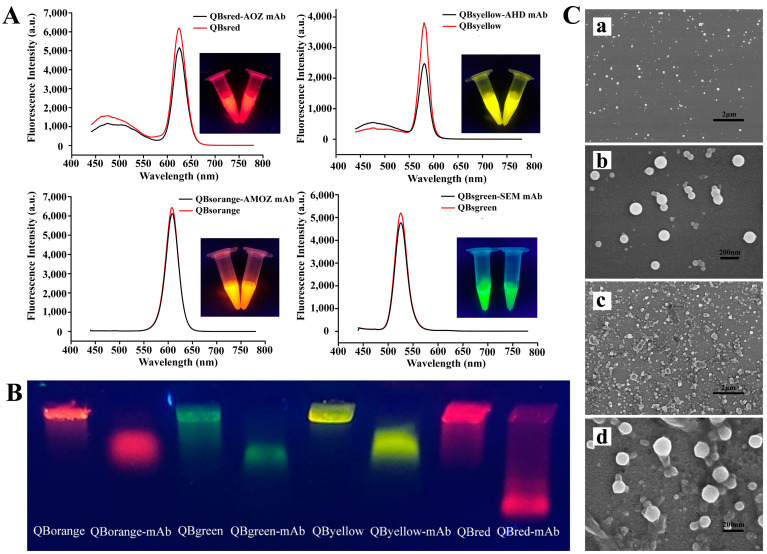
(**A**) Fluorescence emission spectra of QBs and QB-mAb probes. The inset shows the corresponding photograph of QBs and QB-mAb probes solutions under UV light at 365 nm, (**B**) fluorescent images of QBs and QB-mAb in 0.5% agarose gel after gel electrophoresis, (**C**) SEM images of QBs at magnifications of (**a**) 10,000× and (**b**) 60,000×, and QB-mAb probes at magnifications of (**c**) 10,000× and (**d**) 60,000×.

To investigate the successful modification of the mAb on the QBs, different characterization methods were employed. Figure 2A shows the fluorescence spectra of QBs and QB-mAb. After coating with the mAb, the fluorescence intensity of red (625 nm), yellow (575 nm), green (525 nm), and orange (605 nm) QB-mAb probes decreased by 16.81%, 34.64%, 7.77%, and 5.06%, respectively. This was probably because the mAb conjugated on the surface of QBs, reducing the emitted fluorescence signal of the bare QBs. This indicated the successful modification of the mAb on the QBs. Even though the fluorescence intensity decreased, as shown in the inset of Figure 2A, bright red, yellow, green, and orange fluorescence were observed for QB-mAb at the same excitation wavelength of 365 nm. In addition, the quantum yields of the QBs and QB-mAb in different batches were all above 70%. This indicates that the QB-mAb probes have significant application potential for multiplex diagnostic techniques with different signal channels.

Ultraviolet absorption spectra, gel electrophoresis, and dynamic light scattering (DLS) analyses of QBs and QB-mAb were also performed and compared. As shown in Figure S2A, the new characteristic absorption peak at 260 nm of mAb can be found in the QBs’ spectra after conjugation to mAb. It can be inferred that the QB-mAb conjugates were successfully formed. Gel electrophoresis analysis confirmed this result. As shown in Figure 2B, the conjugates and free QBs were transported at different rates through the gel. The difference in the migration rate may be due to the alteration in the size and charge before and after coupling. Compared to those of QBs, the sizes of QB-mAb conjugates increased and the negative surface charges decreased, which resulted in the slower migration of QB-mAb conjugates. After conjugation, the DLS results shown in Figure S2B show that the hydrodynamic sizes of red, yellow, green, and orange QBs increased from 67 nm, 110 nm, 104 nm, and 143 nm to 83 nm, 209 nm, 164 nm, and 191 nm, respectively, with the polydispersity indexes below 0.1, showing narrow and uniform size distribution.

The morphologies of the QBs and QB-mAb probes were examined by scanning electron microscopy. Figure 2(Ca,Cb) show SEM images of the surface of the bare QBs at magnifications of 10,000× and 60,000×, respectively. The QBs were spherical and homogeneous, with a diameter of approximately 100 nm. As shown in Figure 2(Cc,Cd), after coating with mAb, the surface of QB-mAb became rough and had adhesive properties, which confirmed the conjugation of mAb to the QBs.

### 2.3. Optimization of the QB-Based mICA Strip

**Figure 3:**
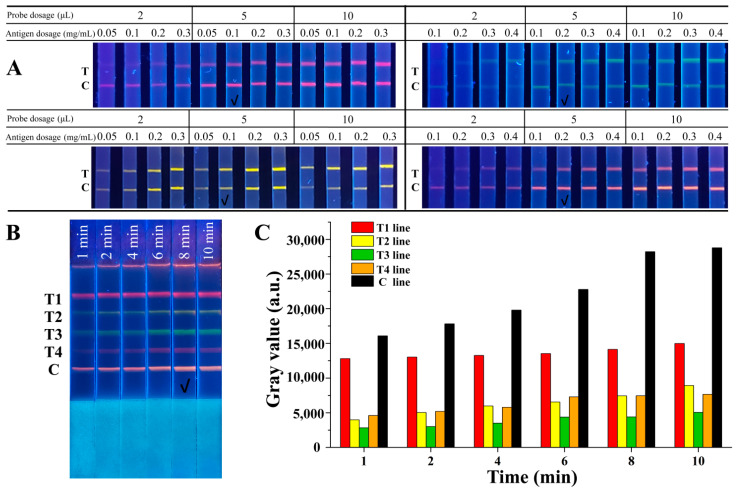
(**A**) Optimization of the concentrations of competitive antigen on T lines and amounts of QB-mAb probes, (**B**) optimization of immunoreaction time, and (**C**) gray values analysis of T and C lines. T1, T2, T3, T4, and C lines were 2-NPAOZ-BSA, 2-NPAHD-BSA, 2-NPSEM-BSA, 2-NPAMOZ-BSA, and rabbit anti-mouse IgG, respectively. The check mark indicated the optimal condition.

To achieve better analytical performance of the QB-based mICA strip, several key parameters were optimized systematically, including concentrations of the competitive antigen on the test lines, amounts of QB-mAb probes, and immunoreaction time. Figure 3A shows the dependence of the fluorescence intensity of the T lines on different concentrations of competitive antigens and the amounts of QB-mAb probes. Strong fluorescence signals were observed when the volume of the QB-mAb probes increased from 2 μL to 5 μL. When the volume was increased to 10 μL, the fluorescence signals did not change significantly in any of the bands. At the same volume of the QB-mAb probes, increasing the concentration of antigens from 0.05 to 0.3 mg/mL increased the fluorescence intensity on T lines. Considering the sensitivity of the mICA strip and cost savings, the fluorescence of the test lines should not be too defined. Therefore, the optimal combinations of usage volume of QB-mAb probes and concentration of coating antigen on test lines were as follows: 5 μL of red QBs probe and 0.1 mg/mL of 2-NPAOZ-BSA on test line for AOZ detection, 5 μL of yellow QBs probe and 0.1 mg/mL of 2-NPAHD-BSA on test line for AHD detection, 5 μL of green QBs probe and 0.2 mg/mL of 2-NPSEM-BSA on test line for SEM detection, and 5 μL of orange QBs probe and 0.2 mg/mL of 2-NPAMOZ-BSA on test line for AMOZ detection, respectively.

The immunoreaction time was optimized, and the results are shown in Figure 3B. The fluorescence intensity of both the T and C lines gradually increased with increasing immunoreaction time. All bands showed sufficiently defined fluorescence signals for visual discrimination at 8 min of running time. The gray values analysis, as shown in Figure 3C, also exhibited an increasing trend before 8 min and then stabilized. Thus, 8 min was chosen as the optimal immunoreaction time.

### 2.4. Performance Evaluation of Multicolor ICA

#### 2.4.1. Sensitivity

**Figure 4:**
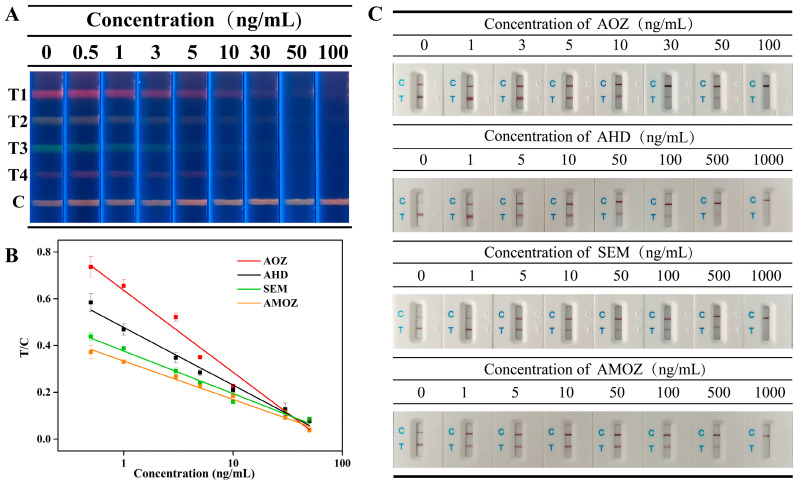
(**A**) Fluorescent image of mICA strip under UV light for detection of AOZ (T1), AHD (T2), SEM (T3), and AMOZ (T4), (**B**) calibration curves of analytes, and (**C**) Commercial colloidal gold test strips for analyte detection.

Sensitivity was evaluated by the cut-off values, and the cut-off values of the multiplex fluorescent lateral flow immunoassay were obtained by analyzing a series of mixtures of nitrofuran metabolites at different concentrations. As shown in Figure 4A, the fluorescence intensity of each corresponding T line gradually decreased as the concentration of nitrofuran metabolites increased. In addition, the fluorescent signal on the corresponding T line completely disappeared when the concentrations of nitrofuran metabolites, including AOZ, AHD, SEM, and AMOZ, all reached 50 ng/mL. This indicated that the cut-off values of our proposed method were 50 ng/mL for AOZ, AHD, SEM, and AMOZ.

Commercial test strips based on colloidal gold immunochromatography were tested under the same conditions, and the results are shown in Figure 4C. The results showed that the cut-off values of commercial test strips were 100 ng/mL, 1000 ng/mL, 1000, and 1000 ng/mL, which were 2-fold, 20-fold, 20-fold, and 20-fold greater than those of our method for AOZ, AHD, SEM, and AMOZ, respectively. Therefore, the cut-off values in our study were less than those obtained using commercial test strips. This indicates that our proposed method is more sensitive than previous methods.

To further detect the target analytes quantitatively, the gray value, which was extracted from the images of the test strip, was introduced in this study. The T/C values of the bands representing different analytes were plotted along with the concentrations of the corresponding analytes, and the calibration curves are shown in Figure 4B. Results showed that the concentration of analytes and the T/C values displayed a good linear correlation between 0.5~50 ng/mL for AOZ, AHD, SEM, AMOZ. The linear equations are presented in Table S1. According to the linear equations, the IC50 values of each analyte were calculated to be 1.80, 1.72, 1.22, and 2.28 ng/mL for AOZ, AHD, SEM, and AMOZ, respectively. Moreover, the calculated LOD values of AOZ, AHD, SEM, and AMOZ were 0.198, 0.138, 0.071, and 0.157 ng/mL, respectively. Compared to traditional quantitative analysis methods for test strips that require professional test strip readers, our method can provide a more objective quantitative detection based on the gray values extracted from test strip images without using specialized instruments. In addition, a comparison of this and previously published methods was performed. As shown in Table S2, the proposed method provides lower or similar LODs.

#### 2.4.2. Cross-Reaction and Specificity

**Figure 5:**
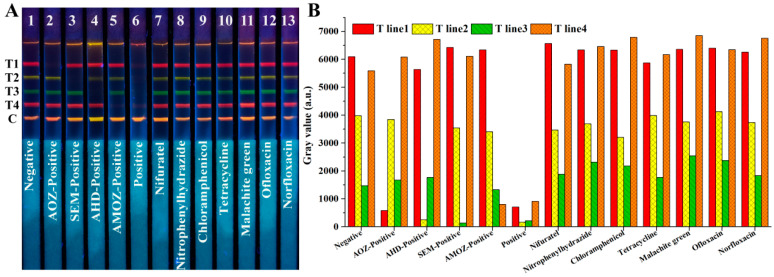
Cross-reaction and specificity analysis of our developed QB-based mICA strip, (**A**) fluorescent image of mICA strip under UV light, (**B**) gray values analysis of four T lines. T1, T2, T3, T4, and C lines were 2-NPAOZ-BSA, 2-NPAHD-BSA, 2-NPSEM-BSA, 2-NPAMOZ-BSA, and rabbit anti-mouse IgG, respectively.

To assess the cross-reaction, AOZ, AHD, SEM, AMOZ, and a mixture of the above four analytes were tested using test strips. As shown in Figure 5A, based on test strips No. 1–6, one may observe that only the fluorescence signals of the corresponding band disappeared in the presence of the corresponding target analytes, while four fluorescent bands disappeared simultaneously in the presence of the four analytes. This indicated that there was no cross-reaction of the developed test strips among these four analytes.

Specificity was investigated by detecting analogues and other fishery drugs, including nifuratel, nitrophenylhydrazide, chloramphenicol, tetracycline, malachite green, ofloxacin, and norfloxacin at a high concentration of 1 μg/mL. As shown in Figure 5A, based on test strips No. 7–13, there was no fluorescence disappearance in the presence of the other analogues or fishery drugs, even at high concentrations. These results indicate that the developed QBs-mICA has excellent selectivity against AOZ, AHD, SEM, and AMOZ. The gray values analysis shown in Figure 5B indicated the same result.

#### 2.4.3. Accuracy and Precision

The accuracy and precision of our QB-based mICA strip were evaluated by detecting blank fish samples spiked with four nitrofuran metabolites separately at three levels (0.5, 5, and 50 ng/mL). The quantitative analysis results were calculated according to the gray values of the test strip images, as mentioned above. As listed in Table S3, the recoveries of four nitrofuran metabolites, including AOZ, AHD, SEM, and AMOZ, at different spiked levels were in the ranges of 82.0–93.3%, 84.2–92.2%, 84.9–92.0%, 90.3–93.0%, respectively, with RSD values less than 10%, respectively. This indicates that the proposed method is suitable for quantitative analysis with good accuracy and precision.

### 2.5. Application in Real Samples

To demonstrate the feasibility of the proposed method for the detection of nitrofuran metabolites in different aquatic products, the QB-based mICA strip was applied to four different samples: red drum, grass carp, shrimp, and scallop. As summarized in Table S4, our test strips could display different intensities of fluorescence bands when they detected real samples with various contamination levels of target analytes. These results confirm that the sample matrices do not have an observed effect on the developed mICA strip.

### 2.6. Conceptual Products for On-Site Detection

**Figure 6:**
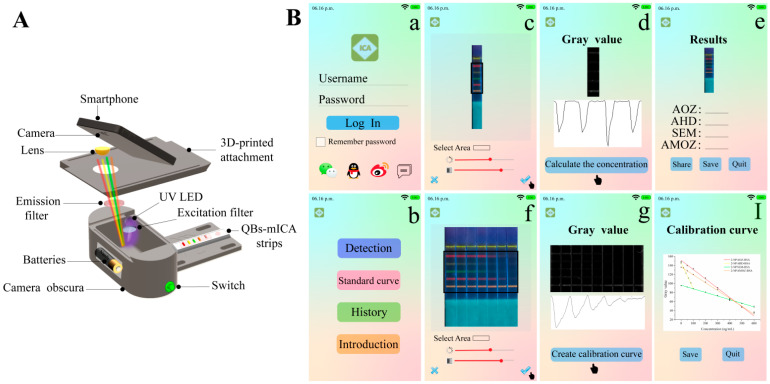
(**A**) The internal structure of smartphone-based immunochromatography reader, and (**B**) the interfaces of analysis smartphone application.

To achieve rapid on-site detection, we designed a smartphone-based immunochromatography reader and developed an analysis application that could rapidly quantify the results [21,22]. As shown in Figure 6A, we used our smartphone camera to directly capture the fluorescent images of the strips using a small and portable reader. As shown in Figure 6B, the application converted fluorescent images to grayscale images. Then, the calibration curves and analyte concentrations were obtained by analyzing the gray values. Traditional fluorescent strip readers, which can read multiple fluorescent bands, must be installed in multiple signal-receiving channels at different wavelengths, which is complicated and expensive. This limits the development of multiplex immunochromatographic techniques for on-site detection. Therefore, compared to the traditional fluorescent strip reader, the portable smartphone-based reader that we conceptually designed in our study shows great potential for rapid on-site detection.

## 3. Materials and Methods

### 3.1. Materials

Carboxyl functionalized CdSe/ZnS quantum dot nanobeads (red QBs, yellow QBs, green QBs, and orange QBs) were purchased from Shanghai Kundao Biotech (Shanghai, China). Rabbit anti-mouse IgG antibodies, anti-2-NPAOZ mAb, anti-NPAHD mAb, anti-2-NPSEM mAb, anti-2-NPAMOZ mAb, 2-NPAOZ-BSA, 2-NPAHD-BSA, 2-NPSEM-BSA, 2-NPAMOZ-BSA were purchased from Shangdong Landu Biotech (Shangdong, China). Nifuratel, nitrophenylhydrazide were purchased from Bide Medical Technology Co., Ltd. (Shanghai, China). Chloramphenicol (CAP), tetracycline (TC), malachite green (MG), ofloxacin (OFL), norfloxacin (NOR), N-hy-droxysuccinimide (NHS), carbodiimide (EDC), 2-(4-morpholino)ethanesulfonic acid (MES), bovine serum albumin (BSA), and other chemicals were purchased from Aladdin-Reagent Co. (Shanghai, China). Sample pads, nitrocellulose membranes, absorbent pads, and polyvinyl chloride backing card were purchased from Shanghai Kinbio Tech Co., Ltd. (Shanghai, China).

### 3.2. Preparation of QB-mAb Probes

Probes that were used for detection were prepared using a modified EDC-mediated activated ester method based on previously reported methods [23,24]. The 2-NPAOZ mAb was labeled with red QBs and the labeling process was as follows: 2 μL of 10 mg/mL red QBs were diluted 10 times with 50 mM MES buffer (pH 6.0) and ultrasonicated for 1 min. To activate the carboxyl groups on the surface of red QBs, the freshly prepared cross linker 10 μL of EDC (1 mg/mL) and 10μL of NHS (2 mg/mL) were added into the red QB solutions, respectively, and mixed well by vortex. The above mixtures were incubated at room temperature in the dark for 30 min on a shaker with vigorous stirring. Subsequently, the activated red QBs were mixed with 5μL of 1 mg/mL 2-NPAOZ mAb and then shaken at 200 rpm for 2 h at room temperature. The mixture was centrifuged for 20 min at 10,000 rpm. The supernatant was removed, and the precipitate was resuspended in 500 μL of MES buffer. The red QB-mAb probes were stored at 4 °C until use.

The yellow, green, and orange colors of the QBs were conjugated to 2-NPAHD mAb, 2-NPSEM mAb, and 2-NPAMOZ mAb, respectively, and the corresponding QB-mAb probes were prepared using the same methods as described above

### 3.3. Characterization of QB-mAb Probes

The morphologies of the QBs and QB-mAb were analyzed by scanning electron microscopy (SEM). SEM was performed using an SU8020 system (Hitachi, Japan) with an operating voltage of 3 kV. UV-visible absorption spectra of QBs, QB-mAb, and mAb were measured using a UV-2550 spectrophotometer (Shimadzu, Japan). The hydrodynamic diameters of QBs and QB-mAb were measured using a Nano-Zs90 dynamic light scattering (DLS) system (Malvern, UK). Fluorescence spectra were recorded using an F-7000 fluorescence spectrophotometer (Hitachi, Japan) under 420 nm excitation. The gel electrophoresis experiment was performed with 0.5% (*w*/*v*) agarose gel at 180 V and 180 mA for 2 h to further confirm the successful formation of the QB-mAb conjugates. 1×Tris Acetate-EDTA buffer (TAE, pH 8.3) was used as the electrophoretic buffer.

### 3.4. Preparation of the QB-Based mICA Strip

As illustrated in Figure S1, our designed QB-based multiplex immunochromatographic strip consisted of a sample pad, an NC membrane, an absorbent pad, and a polyvinyl chloride (PVC) backing card. The sample pad was pretreated by immersing it in PBS solution containing 2% sucrose, 0.5% BSA, and 0.05% Tween-20 for 10 min, and then dried at 37 °C overnight. Target analyte–BSA conjugates, including 2-NPAOZ-BSA, 2-NPAHD-BSA, 2-NPSEM-BSA, 2-NPAMOZ-BSA, and rabbit anti-mouse IgG were dispersed on the NC membrane acting as T1, T2, T3, T4, and C lines, respectively, at a dispensing rate of 1 μL/cm. The interval between the lines was 3 mm. The NC membrane was allowed to dry at 37 °C for 2 h. After that, the sample pad, NC membrane, and absorbent pad were attached to a PVC backing cad and then cut into 3.5 mm wide strips. The strips were stored at room temperature until further use.

### 3.5. Simultaneous Detection of AOZ, AHD, SEM, and AMOZ

The analyte solutions were diluted with PBS to different concentrations. Then 100 μL of analyte solutions were incubated with mixed QB-mAb probes containing 5 μL red QB-mAb probe, 5 μL yellow QB-mAb probe, 5 μL green QB-mAb probe and 5 μL orange QB-mAb probe for 5 min. Subsequently, a strip was inserted into each solution. The probes in the solution flowed from the sample pad to the absorbent pad by capillary force and then reacted with the coating antigens on the test lines. Each strip was removed from the solution after 8 min and placed under a 365 nm UV lamp to observe the fluorescent signals of the test lines and control line.

### 3.6. Performance Evaluation of mICA Strip

The performance of the mICA strip was evaluated in terms of sensitivity, specificity, accuracy, and precision.

The sensitivity of the mICA strip was evaluated by the cut-off values, half-maximal inhibitory concentration (IC50), and limit of detection (LOD). The cut-off values were defined as the lowest concentration that might cause the test lines’ fluorescence to disappear completely. IC50 was defined as the analyte concentration resulting in 50% inhibition of the signal. The LOD was defined as the analyte concentration that resulted in 10% inhibition (IC10) of the signal. The target analytes were diluted with PBS to create a series of solutions at different concentrations for sensitivity evaluation.

Specificity was evaluated by detecting analogues (nifuratel, nitrophenylhydrazide) and other fishery drugs (chloramphenicol, tetracycline, malachite green, ofloxacin, and norfloxacin) at a concentration of 1 μg/mL. In addition, each analyte was detected separately using mICA strips to prove that there was no cross reaction between the four test lines.

The accuracy and precision of the mICA strip were verified by analyzing the recovery and relative standard deviation (RSD).

### 3.7. Real Sample Analysis

Red drum (*Sciaenops ocellatus*), grass carp (*Ctenopharyngodon idella*), shrimp, and scallops (Pectinidae) were purchased from a local supermarket in Jinzhou, China. All samples were packaged foods and stored frozen at the time of purchase. The sample preparation method is according to a previously reported method [11]. The 1 g muscle tissues of the testing samples were minced and hydrolyzed with 1 mL HCl (1 mol/L), and then 200 μL 2-NBA (50 mM) was added for derivatization. The samples were then extracted 3 times with 5 mL ethyl acetate. The extracted solution was centrifuged at 5000 rpm for 20 min at 4 °C. The ethyl acetate fraction was collected and dried under a nitrogen atmosphere. The residue was resuspended with 2 mL of hexane and 1 mL of PBS (0.1 M, pH 7.4). After centrifuged at 6000 rpm for 10 min, the extraction was reconstituted in 1 mL PBS solution and then tested for immunoassay. Spiked samples were prepared by adding standard target analytes.

### 3.8. Data Analysis

Fluorescence images of test strips were collected by a smartphone and then further processed with Image J software to extract the gray values of the T and C lines. The calibration curves were constructed by plotting the T/C values of the bands representing different analytes along with the concentrations of the corresponding analytes.

## 4. Conclusions

In summary, we successfully achieved the simultaneous detection of four nitrofuran metabolites using our new multicolor ICA platform based on QB probes with different emission colors. Taking advantage of the QB probes, the visual detection limits of our method increased by factors of 2, 20, 20, and 20 for AOZ, AHD, SEM, and AMOZ detection, respectively, compared to those of the method with traditional colloidal gold test strips. Furthermore, this method showed reliable specificity, accuracy, and reproducibility, and was applicable to different matrices. Moreover, the gray values of fluorescent bands on the test strip showed a linear relationship with the concentration of analytes at the range of 0.5~50 ng/mL, which indicates that quantitative analysis can be simply performed based on the gray values without any special instrument. Compared to traditional monochromatic immunochromatography, our multicolor method can detect four nitrofuran metabolites more rapidly, sensitively, and intuitively and has the potential for naked-eye detection or a combination with smart readers, such as smartphones and other smart devices.

## Data Availability

The data presented in this work are available in the article and Supplementary Materials.

## Supplementary Materials

The following supporting information can be downloaded at: https://www.mdpi.com/article/10.3390/molecules27238324/s1, Figure S1: Schematic illustration of the QB-based mICA; Figure S2: (A) UV-Vis absorption spectra of mAb, QBs, and QB-mAb probes (B) DLS analysis of QBs and QB-mAb probes; Table S1: Linear equations based on mICA analysis for the nitrofuran metabolites; Table S2: Assay performance comparisons of the published methods; Table S3: Recoveries of QB-based mICA strip for the detection of nitrofuran metabolites; Table S4: Application of QB-based mICA strip to detect nitrofuran metabolites in different samples.

## Sample Availability

Samples of the compounds are not available from the authors.
