# Supplementary material for: Quantum Dot Nanobeads as Multicolor Labels for Simultaneous Multiplex Immunochromatographic Detection of Four Nitrofuran Metabolites in Aquatic Products
Source: Molecules. 2022 Nov 29;27(23):8324. doi: 10.3390/molecules27238324 (PMC9737793; doi:10.3390/molecules27238324)
Supplement: Supplementary file 1 [file molecules-27-08324-s001.zip › molecules-2031797-supplementary.pdf]

Supplementary Material

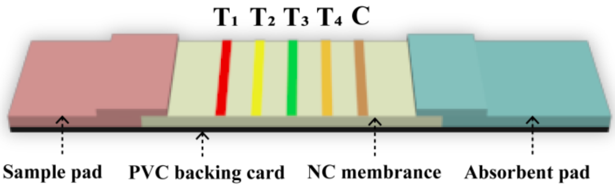

Figure S1. Schematic illustration of the QB-based mICA.

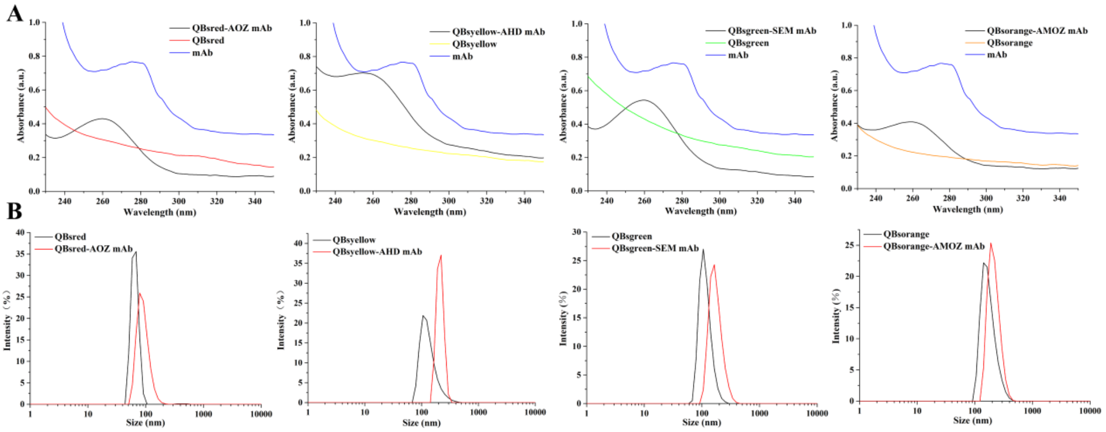

Figure S2. (A) UV-Vis absorption spectra of mAb, QBs and QB-mAb probes. (B) DLS analysis of QBs and QB-mAb probes.

Table S1. Linear equations based on mICA analysis for the nitrofuran metabolites.

Analyte	Standard curve	R^2
AOZ	y= - 0.350 logx + 0.636	0.970
AHD	y= - 0.247 logx + 0.478	0.984
SEM	y= - 0.182 logx + 0.377	0.979
AMOZ	y= - 0.164 logx + 0.333	0.990

Table S2. Assay performances comparison of the published methods.

Analytes	Methods	Samples	LOD	References
AHD	SERS	Sea cucumber	5 ng/g	Fan et al., 2021
AHD	ELISA	Water	0.2 ppb	Liu et al., 2007
AOZ	Immunoassay	Milk powder/Shrimp	0.43 ng/mL	Su et al., 2021
AMOZ	ELISA	Shrimps	0.16 μg/kg	Pimpitak et al., 2009
AMOZ	Immunosensor	Food samples	1 ng/mL	Jin et al., 2011
SEM	HPLC	Food matrices	0.4 μg/kg	Li et al., 2014
AOZ	Multiplex ICA	Red drum/Grass carp/Shrimp/ Scallop	0.198 ng/mL	This work
AHD			0.138 ng/mL	
SEM			0.071 ng/mL	
AMOZ			0.157 ng/mL	

Table S3. Recoveries of QB-based mICA strip for the detection of nitrofuran metabolites.

Sample	Spiked (ng/mL)	Measured (ng/mL)	Recovery (%)	RSD (%)
AOZ	0.5	0.43	86.2	5.86
	5	4.67	93.3	0.49
	50	40.99	82.0	8.84
AHD	0.5	0.44	88.6	6.71
	5	4.61	92.2	3.84
	50	42.12	84.2	9.33
SEM	0.5	0.46	92.0	3.69
	5	4.44	88.7	1.21
	50	42.43	84.9	5.64
AMOZ	0.5	0.46	91.9	7.52
	5	4.52	90.3	2.13
	50	46.49	93.0	5.40

Table S4. Application of QB-based mICA strip to detect nitrofuran metabolites in different samples.

sample	AOZ		AHD		SEM		AMOZ		
	Spiked (μg/kg)	mICA	Spiked (μg/kg)	mICA	Spiked (μg/kg)	mICA	Spiked (μg/kg)	mICA	mICA images
Red drum	0.5	+,+,+[a]	0.5	+,+,+	0.5	+,+,+	0.5	+,+,+	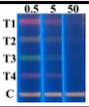
	5	±,±,±[b]	5	±,±,±	5	±,±,±	5	±,±,±	
	50	−,−,−[c]	50	−,−,−	50	−,−,−	50	−,−,−	
Grass carp	0.5	+,+,+	0.5	+,+,+	0.5	+,+,+	0.5	+,+,+	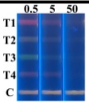
	5	±,±,±	5	±,±,±	5	±,±,±	5	±,±,±	
	50	−,−,−	50	−,−,−	50	−,−,−	50	−,−,−	
shrimp	0.5	+,+,+	0.5	+,+,+	0.5	+,+,+	0.5	+,+,+	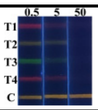
	5	±,±,±	5	±,±,±	5	±,±,±	5	±,±,±	
	50	−,−,−	50	−,−,−	50	−,−,−	50	−,−,−	
scallop	0.5	+,+,+	0.5	+,+,+	0.5	+,+,+	0.5	+,+,+	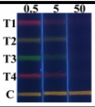
	5	±,±,±	5	±,±,±	5	±,±,±	5	±,±,±	
	50	−,−,−	50	−,−,−	50	−,−,−	50	−,−,−	

[a] Negative result. An obvious fluorescence band was observed.

[b] Weakly positive result. The fluorescence band was light.

[c] Positive result. No fluorescence band was observed.
